# Polarization-Independent Nematic Liquid Crystal Phase Modulators

**DOI:** 10.1021/acsphotonics.5c00098

**Published:** 2025-05-05

**Authors:** Linpei Xue, Steve J. Elston, Stephen M. Morris

**Affiliations:** Department of Engineering Science, 6396 University of Oxford, Parks Road, Oxford, OX1 3PJ, United Kingdom

**Keywords:** nematic liquid crystals, phase modulator, spatial light modulator, optical phase measurements, polarization independent, super twisted nematic liquid crystals

## Abstract

In this paper, we investigate the potential of achieving polarization independent phase modulation using supertwisted nematic (STN) liquid crystal (LC) devices. Here, we describe the use of a burst driving voltage applied to a 180° STN LC device to obtain a twist symmetric H (T-Hs) state, which enables simultaneous modulation of light for all polarizations, demonstrating a polarization independent characteristic in the time domain. Additionally, we consider a 90° twisted nematic (TN) LC device for comparison, as this can also exhibit polarization independent characteristics. Simulations were carried out using a numerical model based on the Ericksen–Leslie continuum equations, which was employed in conjunction with the Jones calculus to simulate the optical properties of the device. The time-dependent optical phase modulation of the device was subsequently measured by using a phase-shifting Mach–Zehnder interferometer. The experimental results demonstrate that an STN device with an 8.9 μm thick LC layer operating in the T-Hs state exhibited a π/2 optical phase modulation in 1 ms for a burst voltage of 30 Vrms that was found to be independent of the incident polarization. These measurements were obtained at room temperature in a single optical path configuration and were found to be in good agreement with the results from the simulations.

## Graphical abstract

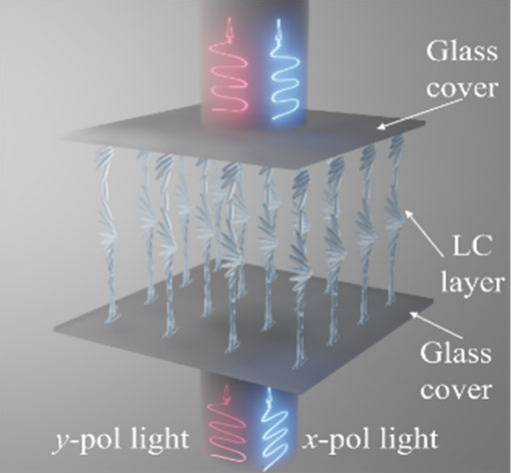

## 1 Introduction

Spatial light modulators (SLMs) are crucial components in modern optics and photonics, offering precise and dynamic control over the properties of light such as the phase, amplitude, and polarization. By exploiting the unique electro-optic properties of liquid crystals (LCs), LC-based SLMs enable real-time manipulation of incident light with incredible flexibility and precision, shaping optical wavefronts for a variety of applications including holography, microscopy, beam shaping, and optical communication systems.^1–3^ Notably, SLMs that are capable of high frame rates, analogue 2π phase modulation, and low driving voltages are in high demand.

To further improve the versatility and efficiency of optical systems, polarization independence is a highly desirable feature in the design and application of LC-based SLMs as it ensures consistent performance in real-world scenarios, where the incident light may exhibit unpredictable or changing polarization characteristics. Polarization independent SLMs would allow incident light to pass through the device without changing the polarization state, eliminating the need for additional optical components, such as polarizers, to manage varying polarization states. This streamlines the optical system, enhances the optical efficiency, and reduces the financial cost.

Broadly, three different techniques have been considered for polarization independent performance using LC-based devices. The first technique is to configure the surface alignment of the LC device at the two opposing substrates to be orthogonal to one another, as exemplified by Twisted Nematic (TN)-LC devices, enabling polarization independence to be achieved while maintaining optical phase modulation with an applied voltage.^4–6^ In a TN-LC device, the LC director near the substrate surfaces aligns parallel to the surface alignment, while in the bulk, the director twists uniformly by 90° due to the boundary conditions in the absence of an applied voltage. When a voltage is applied, the LC director in the middle of the layer orients along the electric field direction, while the director near the substrate surfaces maintains alignment with the surface rubbing direction. At high voltages, the two boundary layers can be regarded as decoupled from the bulk and are oriented along perpendicular directions, allowing for simultaneous optical phase modulation for orthogonal linear polarizations. TN-LC devices have been applied for the purposes of amplitude modulation, phase-only modulation,^7^ and complex modulation. The latter enables simultaneous control of both the amplitude and phase.^8^

Second, polarization independence can be achieved by utilizing certain LC phases, such as blue phase LCs (BPLC),^9,10^ polymer-stabilized blue phase LCs (PS-BPLCs),^11–13^ short pitch chiral nematic LCs (CLCs),^14–17^ and polymer-dispersed LCs (PDLC).^18–20^ BPLCs exhibit a three-dimensional periodic lattice structure with spontaneous chirality and are optically isotropic, enabling uniform optical modulation of light for all polarization states. Additionally, BPLCs exhibit major advantages in terms of millisecond response times, simple fabrication methods (no need for surface alignment layers), a large optical Kerr effect, and the ability to perform continuous optical phase modulation, making them a promising candidate for SLM technologies. Toward this end, previous research proposed optimizing the material parameters and cell gap of the BPLC device to achieve a full 2π phase shift at a voltage of 26 V.^10^ However, BPLCs typically exhibit limited temperature ranges, which has led to innovations such as cross-linked polymer networks to stabilize the mesophase (PS-BPLCs). Nonetheless, polymer stabilization remains a challenging process and requires a high driving voltage, which increases power consumption.^12^ While reducing the operating voltage is possible by increasing Δε, this approach typically results in a higher viscosity and potentially slower response time. Additionally, the prolonged charging time subsequently limits the device’s resolution and frame rate.^12^ What’s more, PS-BPLC devices exhibit limited phase modulation. For instance, achieving a full 2π phase shift required 24 V_rms_ and a four-pass optical configuration to reach this level of phase modulation.^13^

For CLCs with a pitch smaller than the wavelength of light, the uniform standing helix and focal conic states have been exploited to demonstrate polarization independence owing to their effective birefringence averaging and anisotropic properties.^14^ This attribute arises from the helical macroscopic structures, which possess circular symmetry, enabling uniform interaction with incident light regardless of the polarization state. CLCs have also been extensively applied in the infrared (IR) band as polarization independent variable optical attenuators^15^ and in the THz band as polarization-independent 2π THz phase shifters.^16^ PDLCs, on the other hand, have been used to achieve polarization independence by randomly dispersing the orientation of the LC director, ensuring that incident light of any polarization experiences the same effective refractive index, resulting in a consistent phase shift.^18^ It has been suggested that these structures can be optimized by making the LC droplets pinned near the glass substrates^19^ and using nm-sized PDLCs.^20^ However, PDLCs can suffer from limited phase modulation and unwanted losses due to light scattering.

The third method involves stacking two birefringent layers orthogonally to enable phase modulation in both the *x* and *y* directions independently.^21–23^ Researchers inserted an ultrathin polymer film in the middle of two separate orthogonal LC layers resulting in a polarization-independent phase modulation with a 2π phase shift at 9 V_rms_ and an 8.1π phase shift at 40 V_rms_.^23^ Despite the extensive range of different demonstrations and solutions presented to date, the aforementioned approaches are not without their drawbacks and undesirable properties such as limited optical phase modulation, complicated fabrication procedures and device architectures, and/or the need for specialized LC compounds.

In this work, we present an alternative approach to achieving polarization independent optical phase modulation by applying a burst driving voltage to a 180° supertwisted nematic (STN) LC device to make use of the twist symmetric H (T-Hs) state. STN-LCs, characterized by their greater twist compared to TN-LCs, have also been employed in LC displays^24–26^ and SLM technology for both amplitude modulation and phase modulation. For instance, the use of 180°-twist STN-LC in a π-cell is known to form a π-twist state,^27,28^ which has the advantage of inducing a fast and uniform transition to the bend state without the need for nucleation. This transition exhibits a much lower voltage threshold when compared to that of a conventional nematic pi-cell design. In this work, we use a combination of simulations and experiments to demonstrate that accessing the T-Hs state in a 180° STN-LC using a burst driving voltage can result in polarization-independent phase modulation. For comparison, simulations are also conducted for a 90° TN-LC device, which is capable of polarization independent phase modulation, but suffers from limitations that are not exhibited in the T-Hs state of the 180° STN-LC device.

This Article is structured as follows. With the aid of simulations, we begin by considering the behavior for a range of TN and STN devices with different alignment conditions. Results indicate that both the 90° TN device and the T-Hs state found in the 180° STN planar-aligned LC device have the potential to exhibit polarization independent phase modulation, with the latter showing more promise in terms of device performance. In Section 2, we present the phase modulator concept that exploits the T-Hs state and introduce the practical method used to obtain it, followed by a study of the switching regimes in the 180° STN planar LC device. In Section 3, results are presented from our simulation model that has been used to predict the polarization independent behavior of the LC devices considered in this work. The penultimate section, Section 4, presents experimental results of the phase modulation of the 180°-twist STN planar LC device using a phase-shifting Michelson interferometer to measure the optical phase in the time domain. The measured optical phase modulation of the T-Hs state is then analyzed and compared with results from simulations. Finally, Section 5 concludes the work.

## 2 Phase Modulator Concept

Figure 1 illustrates four different 90° TN/180° STN-LC device configurations with the potential for showing polarization independent characteristics. The director distribution was determined using a methodology that will be described in further detail in Section 3.1. These configurations are based on 90°-twist TN LC devices and 180°-twist STN LC devices. For the 90°-twist TN LC device, the director in the middle of the LC layer is either tilted (referred to as a tilted LC device) or aligned parallel to the surface (referred to as a planar LC device). The 180° STN-LC devices were fabricated by doping a chiral additive to a nematic LC host. By precisely controlling the chiral dopant, the pitch of the LC mixture can be set to be twice the thickness of the LC layer, creating a 180° twist state within the device. By filling the LC mixture into a pi-cell (antiparallel pretilt angles on both surfaces), the director in the middle of the LC layer becomes tilted; referred to herein as a tilted STN device. Conversely, when filled into a Fréedericksz device (parallel pretilt angles on both surfaces), the LC director in the middle of the layer aligns parallel to the surface, we refer to this as a planar STN device.

**Figure 1:**
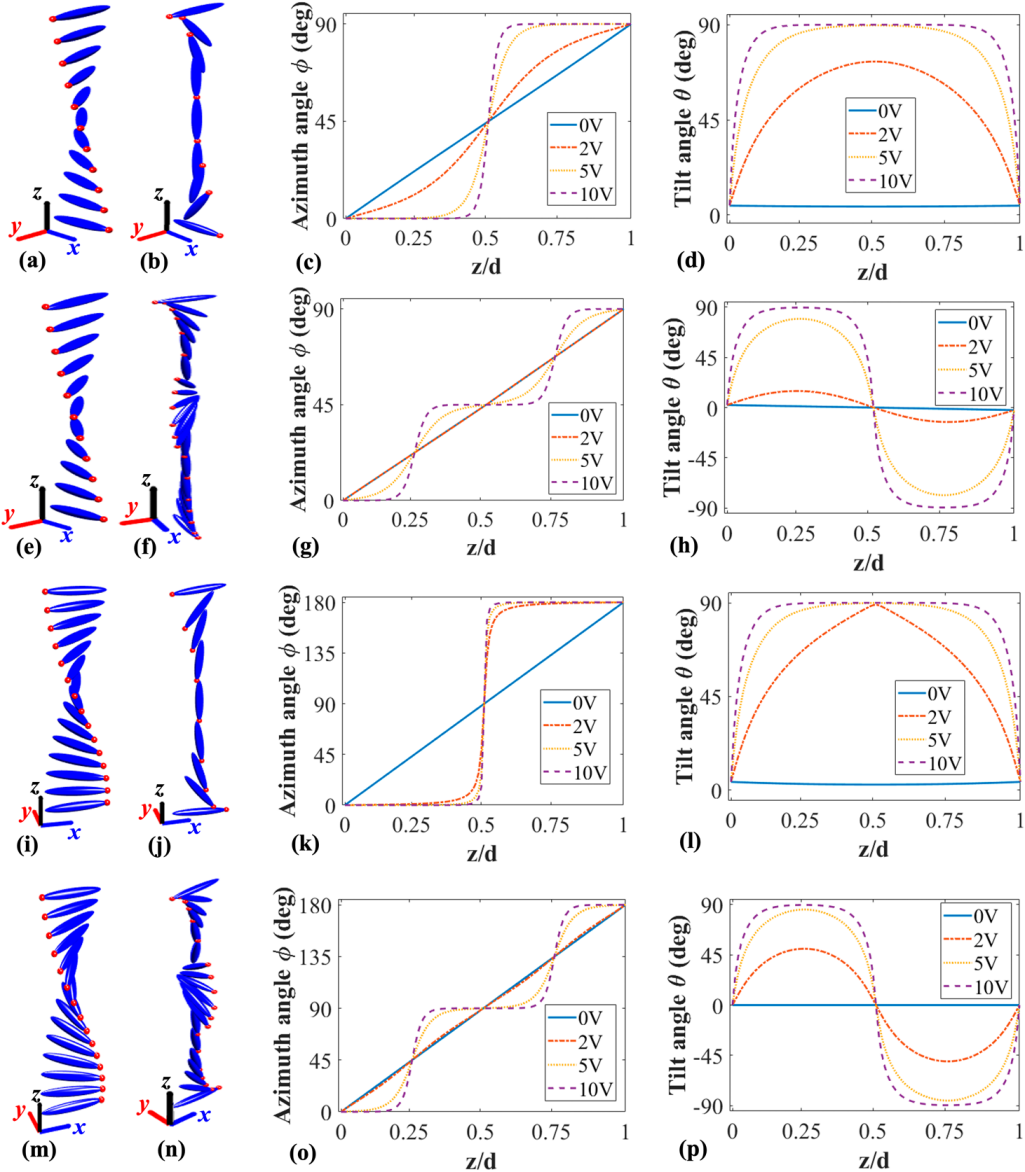
Simulations of the director distribution for the four different LC devices. These include a 90° TN-LC device with the central director tilted relative to the surface plane (tilted 90° TN-LC device), a 90° TN-LC device with the central director parallel to the surface plane (planar 90° TN-LC device), a 180° STN device with antiparallel pretilt alignment (tilted 180° STN-LC device), and a 180° STN device with parallel pretilt alignment (planar 180° STN-LC device). Visualization of the director distribution of the tilted 90° TN-LC device: (a) the ground state with no voltage applied and (b) the twisted vertical state under an applied voltage of 10 V_rms_. (c, d) The azimuth angle (the angle between the projection of the director on the *xy*-plane and *x*-axis) and the tilt angle (the angle between the director direction and the *xy*-plane) of the LC director, respectively, as a function of the position within the LC layer for different applied voltages, showing the formation of 90° twisted vertical state. Visualization of director distribution of the planar 90° TN-LC device under a sudden voltage: (e) the ground state with no voltage applied and (f) the 90° twisted Hs state under a burst driving voltage of 10 V_rms_. (g) and (h) are director profiles under different applied voltages showing the formation of 90° twisted Hs state. Visualization of the director distribution of the tilted 180° STN-LC device: (i) the ground state with no voltage applied and (j) the twisted vertical state under an applied voltage of 10 V_rms_ and (k) and (l) are the azimuth and tilt angles, respectively, under different applied voltages showing the formation of a 180° twisted vertical state. Visualization of the director distribution of the planar 180° STN-LC device under a sudden voltage: (m) the ground state with no voltage applied and (n) the 180° twisted Hs state under a voltage of 10 V_rms_. (o) and (p) are the director profiles under different applied voltages showing the formation of 180° twisted Hs state.

Figure 1(a) – (d) illustrate the change in director configuration for the tilted 90° TN-LC device with and without an applied voltage. Figure 1(a) shows the director profile in the voltage-off state. In this case, when the LC device is placed between parallel polarizers, no light is transmitted at a zero voltage. However, if placed between crossed polarizers, all light passes through, demonstrating a so-called polarization rotation effect. When an electric field above the threshold is applied to the device, the LC director in the bulk tends to reorient in the direction parallel to the applied electric field (as shown in Figure 1(b)), which coincides with the light propagation direction. In this case, the polarization rotation effect gradually diminishes, allowing for light transmission through parallel-aligned polarizers. The evolution in the director profile (in terms of the azimuth and tilt angle) with an increase in the applied voltage is shown in Figure 1(c) and (d). At high voltages, this reorientation induces the same changes in the effective birefringence within two boundary layers along the *x* and *y* directions, leading to a consequential phase shift in the two directions simultaneously. Notably, in this state, the optical characteristics of the outgoing light will not be affected by the LC layer for any initial polarization state of the incoming light, regardless of whether the polarization is along the *x*-axis or *y*-axis. As a result, at high voltage the TN-LC device exhibits polarization independent characteristics.

When a sudden voltage is applied to the planar 90° TN-LC device, whose voltage off state is shown in Figure 1(e), the device can form two extra boundary layers in the middle of the device at 45° relative to the alignment direction, as shown in Figure 1(f). This state is a transient state with a lifetime of around a few tens of milliseconds. The director profiles for the whole process as the voltage increases are shown in Figure 1(g) (azimuth angle) and (h) (tilt angle). Unfortunately, the two extra boundary layers that are formed do not help with polarization independence. However, this configuration motivated us to consider the transient state in the planar 180° STN-LC device, *vide-infra*.

The tilted 180° STN-LC device is commonly used in the design of optical phase modulators, typically using the so-called π-twist state.^27,28^ In the voltage off state, it exhibits a 180° twist of the LC director across the device, as shown in Figure 1(i). When a voltage is applied, the LC director gradually reorients to align with the external electric field direction, forming a bend-like state known as the twisted vertical (T-V) state or π-twist state, as shown in Figure 1(j). In this process, as the LC director begins to untwist, amplitude modulation is achieved when the LC is placed between polarizers. The untwisting results in a change in the effective birefringence of the device, leading to a continuous phase change. The director profiles simulated for this device under applied voltages are shown in Figure 1(k) and (l) for the azimuth and tilt angle, respectively.

Figures 1(m) – (p) illustrate the corresponding behavior of the planar 180° STN-LC device. When we apply a sudden voltage to the LC, it undergoes a distinct transformation, forming two layers in the middle of the device with directions that are oriented perpendicular to the surface alignment, as illustrated in Figure 1(n). This is termed the twisted Hs state (T-Hs). The two boundary layers next to the surface are along the *y*-direction and the newly formed boundary layers in the center of the device are along the *x*-direction. This dual-layer configuration enables the device to simultaneously generate phase modulation in both the *x* and *y* directions, exhibiting a potentially polarization independent performance for a sudden applied voltage. Like the planar TN configuration, this state is also transient, with a lifetime of only a few tens of milliseconds. The director profiles for different applied voltages are depicted in Figure 1(o) and (p). In comparison to the conventional 90° TN-LC device, which forms only one boundary layer in each direction, the planar 180° STN-LC device forms two boundary layers in each direction. As a result, this allows the T-Hs state in the planar 180° STN-LC device to achieve double the phase modulation of that observed for the 90° TN-LC device.

The simulations presented in Figure 1 indicate that the planar180° STN-LC device warrants further investigation in terms of its potential for polarization independent phase modulation. Toward this end, Figure 2(a) illustrates the typical transformations between the different states of a planar180° STN-LC device, similar to the transformations observed in the nematic pi-cell studied previously.^29^ With no applied voltage, the LC director continuously twists 180° within the device, forming the planar twisted-twist (T-T) state with the LC director in the middle of the layer aligned parallel to the surface. When the applied voltage is above a critical voltage V_th_ (TT) (V_th_(TT) ≈ 1.8 V_rms_), the internal electric field is sufficient to switch the device to a twisted asymmetric H (T-Ha) state (E > E_T‑Ha_). The director profile then transforms into one of the T-Ha states, which is analogous to the Ha state in a conventional nematic pi-cell but with the director continuously twisted. Alternatively, a transient twisted Hs (T-Hs) state is obtained if a sudden voltage above a higher threshold voltage (V_th_ (T-Hs)) is applied to the T-T state, then the internal field is above that required to switch to a symmetric state (E > E_T‑Hs_).

**Figure 2:**
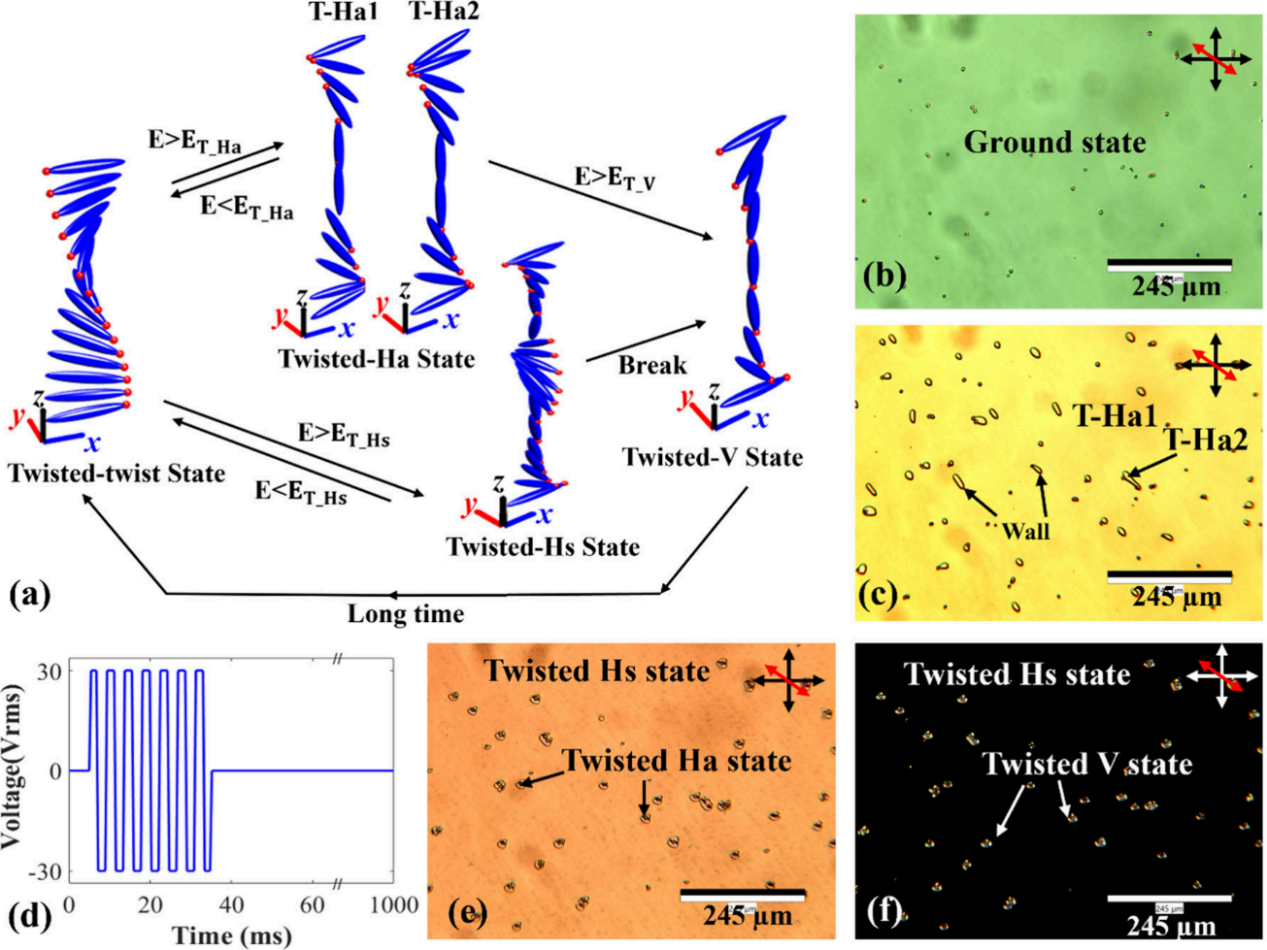
Switching states of a planar 180° STN-LC device. (a) An illustration showing the transition into different states for different applied electric field conditions. Representative polarizing optical microscope images of (b) the twisted-twist (T-T) ground state of the device under no applied voltage (the black dots in the image are the spacer beads distributed throughout the cell to obtain a uniform cell gap); (c) the twisted Ha state formed under a continuous driving voltage of 4 V_rms_; (d) the waveform of the burst driving voltage without modulation used to drive the device to capture the T-Hs state; (e) a mixture of twisted Hs states and twisted Ha states under a burst driving voltage of 3.5 V_rms_ for 90 ms; (f) mixture of twisted Hs states and twisted V states under a burst driving voltage of 10 V_rms_ for 70 ms. The crossed double-headed black arrows (white arrows in (e)) represent the orientations of the transmission axes of the polarizer and the analyzer, and the double-headed red arrows represent the rubbing direction of the alignment layers.

The T-Hs state is a transient state that tends to drift into the T-Ha state(s) or transform to the T-V state directly under a higher applied voltage. When the operating voltage is increased above the threshold for the T-V state, V_th_ (T-V), which is approximately 5.8 V_rms_ for the device under investigation, the internal electric field is increased above that required to switch to a T-V state (E > E_V_), and both the T-Hs and T-Ha states will transform to the T-V state because the T-V state is energetically favorable at high voltages. The T-Hs state can persist for a few milliseconds even under a high voltage exceeding V_th_ (T-V). In this investigation, the device maintained the T-Hs state for approximately 35 ms under an applied voltage of 30 V_rms_. For very high voltages, the T-V state shows very little twist. Moreover, when the voltage is removed and the device is in either the T-Ha or T-Hs state, the director alignment will return to the T-T state directly; on the contrary, when the voltage in the T-V state is removed, the device gradually returns to the T-T state via domain growth over the course of a few seconds.

To obtain the transient T-Hs state in practice, a burst driving method is typically required.^29^ The burst driving voltage consists of an operating period, where the voltage is applied to stimulate the formation of the T-Hs state, and a relaxation period, where no voltage is applied, ensuring that the device fully recovers to the T-T state. The operation period needs to be long enough to swich the device to the T-Hs state but short enough to avoid it collapsing into either the T-Ha state or nucleating into the T-V state. By repeated burst voltage periodicities, a stable T-Hs state can be regularly obtained.

The device used in the experiment consisted of glass substrates that were coated with indium tin oxide (ITO) electrodes and antiparallel rubbed polyimide alignment layers, referred to in this work as a Fréedericksz cell. The surface pretilts were typically 4° to 5°, and the cell gap was 8.9 μm. The LC mixture was prepared by adding the chiral dopant R811 (a low twisting power chiral dopant sourced from Merck KGaA) (a concentration by weight of 0.48 wt %) into the nematic LC mixture, E7 (Synthon Ltd.) so as to form a chiral nematic LC with a long pitch of 17.8 μm. By capillary filling into the Fréedericksz cell, a planar 180° STN-LC device was obtained. In addition to the twist profile due to the chiral dopant, the LC director also exhibits a minor tilt in opposite directions influenced by the pretilt angle for the two different surface alignments. This leads to the director in the middle of the LC layer aligning perpendicular to the surface alignment direction while maintaining a tilt angle of 0°, parallel to the surface. To distinguish the transformation between the states, the device was positioned between crossed polarizers on a polarizing optical microscope (POM) (Olympus BX51), with the rubbing direction (optical axis) aligned 45° to the polarizer and analyzer pair. The applied voltage was a square wave of 1 kHz with a variable burst duration repeated every 1 s.

Figure 2 demonstrates the voltage switching processes in a planar 180° STN-LC device with the aid of exemplar POM images of the various states. In Figure 2b, the device exhibits a twist ground state with no applied voltage. When a continuous driving waveform was applied to the device, the T-Ha and T-V states could be observed for lower and higher voltages, respectively, but the T-Hs could not be observed due to its transient lifetime. Both the T-Ha and T-V states generally exhibit a uniform color in the image. T-Ha states can be distinguished by the presence of domain walls, which appear within the field of view when a voltage was applied, as shown in Figure 2c. These domain walls form due to the opposite reorientation of the LC director in adjacent regions, leading to the formation of two distinct states: T-Ha1 and T-Ha2. The primary difference between these states lies in whether the LC director tilt distortions accumulate predominantly at the top surface or at the bottom surface of the device. This behavior is analogous to the well-known Ha1 and Ha2 states observed in a nematic π-cell.^29^ Despite their structural differences, the T-Ha1 and T-Ha2 states exhibit similar birefringent properties, making them challenging to differentiate based on optical effects alone. The domain wall serves as the interface between these two regions, where the LC director is parallel to the surface in the center of the wall.^30^ In Figure 2c, T-Ha states can be seen as a yellow color with domain walls formed under a continuous driving voltage of 4 V_rms_.

To generate the T-Hs state, a burst driving voltage was applied to the device, resulting in a more complex scenario due to a mix of states arising from imperfect surface alignments. The waveform of the burst driving voltage without modulation is shown in Figure 2d. The image of the T-Hs state was captured during the voltage on operation period, during which the T-Hs state remains stable and, for higher drive voltages, typically exhibits a uniform dark color, as expected for a polarization-independent phase modulator. However, in some cases, when a burst driving voltage with lower amplitude is applied, the T-Hs state appears colored rather than dark, as shown in Figure 2e; this state would not be suitable for polarization independent phase modulation. This phenomenon occurs because a twisted structure remains within the device, and the boundary layers remain coupled, preventing complete polarization independence and leading to color variations in the image. Additionally, a mixture of T-Hs and T-Ha states can form under the burst driving voltage, with T-Ha states appearing around beads or defects due to imperfect surface alignment. However, the T-Ha state consistently collapses during the relaxation period between voltage bursts. When the T-Ha region remains small relative to the total T-Hs area and the T-Hs state maintains its polarization-independent characteristics (appearing dark under POM with crossed polarizers), the device still meets the design requirements for a polarization-independent phase modulator based on the T-Hs state.

As the voltage increases, Figure 2f depicts the mixture of the T-Hs and T-V states for a burst driving voltage of 10 V_rms_ for 70 ms, with the image taken during this 70 ms voltage on period. The T-Hs state appears as the black regions which dominate the image. This is because the T-Hs state exhibits polarization independent characteristics, which means it does not alter the polarization of the incident light after passing through the polarizer on the microscope. Therefore, when the device is placed between crossed polarizers, the image appears black. In addition, the T-V state is also observed in the image, emerging where parts of the T-Hs state have evolved into the T-V state, particularly around the spacer beads. During this stage, the regions of the T-V state are small and fully collapsed to the ground state between bursts, leaving no impact on the T-Hs state formation and phase modulator design. However, if the amplitude and duration of the burst driving voltage are further increased, the T-V states will then dominate and replace all of the T-Hs states, making the device unsuitable for a polarization independent phase modulator.

The above characterization shows that the T-Hs state can be obtained by applying a burst driving voltage to the planar 180° STN-LC device, notwithstanding the potential mixture of various states due to imperfect surface alignment. However, the conditions for using the T-Hs state as a polarization-independent phase modulator are stringent. To ensure an effective polarization-independent phase modulation, several criteria must be met. First, the proportion of the T-Ha states in the mixture of T-Ha and T-Hs states is not detrimental, and the formed T-V state fully collapses during the relaxation between repeated voltage bursts. Additionally, the boundary layers within the device must be decoupled, ensuring that the device exhibits a dark image under the POM with crossed polarizers, which is indicative of polarization independence. When these conditions are satisfied, the device is particularly promising for use in optical phase modulation that requires polarization independent performance. In the following section, results are presented to confirm the polarization independent properties.

## 3 Phase and Amplitude Modulation Simulations

### 3.1 The Model

To demonstrate the intricate dynamics of the T-Hs state, we developed a simulation method to first estimate the transient switching behavior of the LC director before then employing Jones calculus to determine the potential polarization independence and the optical phase modulation performance based on the obtained director profiles.

The transient switching dynamics of the twisted LC device were investigated by using a numerical model designed to solve the Ericksen–Leslie continuum equations, offering insights into the LC director reorientation during transient switching events. The free-energy density considered in the simulations is the composite of elastic, electrostatic, and dielectric contributions, which are the core factors influencing the director behavior. Besides, we deliberately disregard the internal flow within the LC and the chiral factor (*q*), since they have a secondary impact on the calculation results, but their removal can greatly streamline the calculation process.

The general form of the Frank elastic energy density, *f*_d_, can be written in terms of the director **n** as

1 $$f_{d}=\frac{1}{2}K_{11}{\left[\nabla \cdot n\right]}^{2}+\frac{1}{2}K_{22}{\left[n\cdot \left(\nabla \times n\right)\right]}^{2}+\frac{1}{2}K_{33}{\left[n\times \left(\nabla \times n\right)\right]}^{2}$$

where *K*_11_, *K*_22_, and *K*_33_ are the splay, twist, and bend elastic constants, respectively. The electrostatic energy density *f*_e_ takes the following form:

2 $$f_{e}=-\frac{1}{2}{\Delta \varepsilon \varepsilon }_{0}{\left(n\cdot E\right)}^{2}$$

where Δε is the dielectric anisotropy, ε_0_ is the permittivity of free space, and **E** is the internal electric field. We define the director in modified spherical polar coordinates, where the LC orientation is represented by angles θ (the director tilt angle out of the surface plane) and φ (the director twist angle around the *z*-axis). Both are a function of time and position across the LC layer (defined as the *z* coordinate in what follows). In this case, the director can be expressed as

3 n=(cos⁡θ⁡cos⁡φcos⁡θ⁡sin⁡φsin⁡θ)

The applied electric field **E** is along the *z*-direction and the total energy density, *L*, is given by

4 $${L=f}_{d}+f_{e}=\frac{1}{2}K_{11}{\cos^{2}\theta \left(\frac{\partial \theta }{\partial z}\right)}^{2}+\frac{1}{2}K_{22}{\cos^{4}\theta \left(\frac{\partial \varphi }{\partial z}\right)}^{2}+\frac{1}{2}K_{33}{[\sin^{2}\theta \left(\frac{\partial \theta }{\partial z}\right)}^{2}+{\sin^{2}\theta \cos^{2}\theta \left(\frac{\partial \varphi }{\partial z}\right)}^{2}]-\frac{1}{2}{\Delta \varepsilon \varepsilon }_{0}\sin^{2}\theta {E_{z}}^{2}$$

where *E*_*z*_ is the local electric field that is determined from the requirement for continuity in the *z*-component of the electric flux density. To determine the director behavior, we then need to substitute eq 4 into the Euler–Lagrange equations, resulting in two dissipation equations, one for each of the angular deformations θ and φ:

5 $$\frac{\partial }{\partial z}\left(\frac{\partial L}{\partial \theta^{\prime }}\right)-\frac{\partial L}{\partial \theta }=\frac{\partial D}{\partial \mathrm{\theta ̇}}$$

6 $$\frac{\partial }{\partial z}\left(\frac{\partial L}{\partial \varphi^{\prime }}\right)-\frac{\partial L}{\partial \varphi }=\frac{\partial D}{\partial \mathrm{\varphi ̇}}$$

where D is the dissipation function.

The total energy density, *L*, can then be substituted into eqns [Disp-formula eq5] and 6 to give the following:

7 $$\frac{\partial D}{\partial \mathrm{\theta ̇}}={\left(\frac{\partial \varphi }{\partial z}\right)}^{2}\left(K_{33}\sin^{2}\theta -K_{33}\cos^{2}\theta +2K_{22}\cos^{2}\theta \right)\cos\theta \sin\theta +{\left(\frac{\partial \theta }{\partial z}\right)}^{2}\left(K_{33}-K_{11}\right)\cos\theta \sin\theta +\frac{\partial^{2}\theta }{\partial \theta^{2}}\left(K_{11}\cos^{2}\theta +K_{33}\sin^{2}\theta \right)+{\Delta \varepsilon \varepsilon }_{0}\cos\theta \sin\theta {E_{z}}^{2}$$

8 $$\frac{\partial D}{\partial \mathrm{\varphi ̇}}=-2\frac{\partial \varphi }{\partial z}\frac{\partial \theta }{\partial z}\cos\theta \sin\theta \left[2K_{22}\cos^{2}\theta +K_{33}\left(\sin^{2}\theta -\cos^{2}\theta \right)\right]+\frac{\partial^{2}\varphi }{\partial \varphi^{2}}\cos^{2}\theta \left(K_{22}\cos^{2}\theta +K_{33}\sin^{2}\theta \right)$$

which are then solved using a finite difference approach, with a regular grid in the *z*-direction. As noted above, flow components were not considered. Additionally, the LC director tilt is fixed at the surfaces, with the tilt angle specified as 4°, which allows for the computation of θ­(*z*,*t*) and φ­(*z*,*t*).

To determine the optical performance of the TN and STN-LC devices, the Jones calculus was used to extract the optical phase modulation from the obtained director profiles. By considering that the light is traveling along the positive *z*-direction through the LC device, the electric field for the outgoing light, **E**_**out**_, can then be expressed as

9 $$E_{\mathrm{out}}=J_{\mathrm{LC}}E_{\mathrm{in}}$$

where **E**_**in**_ is the electric field of the incident light and **J**_**LC**_ is the LC phase retarder.

To represent the entire LC device, we conceptualize the device, with a thickness of *d*, as being comprised of *N*_*z*_ = 101 distinct slices. Each slice is akin to a plate characterized by specific angles, θ­(*z*,*t*) and φ­(*z*,*t*). The internal 99 slices have a thickness of $\frac{d}{N_{z}-1}$ , while the two surface slices each have a half-thickness of $\frac{d}{2{(N}_{z}-1)}$ to accurately model the surface layer performance. The total optical effect is obtained by multiplying the Jones matrices of all individual slices. The whole LC device can then be expressed as

10 $$J_{\mathrm{LC}}\left(V,t\right)=J_{N_{z}}J_{N_{z}-1}...J_{2}J_{1}=\left[\begin{array}{cc} {Ae}^{i\delta_{x}} & B\\ C & De^{i\delta_{y}} \end{array}\right]$$

where ***J***_**LC**_(*V*,*t*) is the Jones matrix for the entire LC device under an applied voltage *V* at time *t*. **J**_**1**_ and **J**_**N**_**z**__ are two surface slices with a thickness of $\frac{d}{2{(N}_{z}-1)}$ , while **J**_**2**_ to **J**_**N**_**z**_ **–1**_ are the internal slices with a thickness of $\frac{d}{N_{z}-1}$ . *A* and *D* represent the amplitude ratios for the horizontal and vertical polarization components in the output light to those in the input light, respectively, while δ_*x*_ and δ_*y*_ refer to the phase shift for these polarization components. Additionally, *B* and *C* refer to the conversion ratio between one polarization component in the input light to another one in the output light.

The Jones matrix for the surface slices (*n* = 1 or *n* = *N*_*z*_) is expressed as

11 $$J_{n}=R\left(-\varphi \right)MR\left(\varphi \right)=\left[\begin{array}{cc} \cos\varphi & -\sin\varphi \\ \sin\varphi & \cos\varphi \end{array}\right]\left[\begin{array}{cc} e^{2\pi /\lambda n_{\mathrm{eff}}d/{2(N}_{z}-1)} & 0\\ 0 & e^{2\pi /\lambda n_{o}d/{2(N}_{z}-1)} \end{array}\right]\left[\begin{array}{cc} \cos\varphi & \sin\varphi \\ -\sin\varphi & \cos\varphi \end{array}\right]$$

For the internal slices (2 ≤ *n* ≤ *N*_*z*_ – 1), the Jones matrix is

12 $$J_{n}=R\left(-\varphi \right)MR\left(\varphi \right)=\left[\begin{array}{cc} \cos\varphi & -\sin\varphi \\ \sin\varphi & \cos\varphi \end{array}\right]\left[\begin{array}{cc} e^{2\pi /\lambda n_{\mathrm{eff}}d/N_{z}-1} & 0\\ 0 & e^{2\pi /\lambda n_{o}d/N_{z}-1} \end{array}\right]\left[\begin{array}{cc} \cos\varphi & \sin\varphi \\ -\sin\varphi & \cos\varphi \end{array}\right]$$

The effective birefringence, *n*_eff_, is given by

13 $$n_{\mathrm{eff}}=\frac{n_{e}n_{o}}{\sqrt{n_{e}^{2}\sin^{2}\theta +n_{o}^{2}\cos^{2}\theta }}$$

where *n*_o_ and *n*_e_ are the ordinary and extraordinary refractive indices, respectively. The angles θ­(*z*,*t*) and φ­(*z*,*t*) define the orientation of the LC director within each layer, contributing to the overall transmission properties. θ­(*z*,*t*) controls the optical phase delay, while φ­(*z*,*t*) determines the rotation angle.

The transformation of polarization states as light passes through the device can be expressed as

14 (Ex_o(V,t)Ey_o(V,t))=[AeiδxBCDeiδy](Ex_iEy_i)

where *E*_*x*_*o*_ and *E*_*y*_*o*_ are the electric fields for the outgoing light for the *x* and *y* polarizations, respectively, and *E*_*x*_*i*_ and *E*_*y*_*i*_ are the electric fields for the incoming light for *x* and *y* polarizations, respectively.

The optical phase at various time intervals or applied voltages can be extracted from the matrix expression in eq 14. This enables us to determine the dynamic phase modulation, offering valuable insights into the dynamic behavior of the LC devices under different operating conditions. Notably, the elements of the leading diagonal in the matrix of **J**_**LC**_(*V*,*t*) represent the amplitude and phase changes for light that remains polarized in the same direction as the incident light. On the other hand, the off-diagonal elements of the Jones matrix **J**_**LC**_(*V*,*t*) denote the amplitude and phase changes for light that becomes polarized in a direction orthogonal to the incident light. Therefore, for an ideal polarization-independent LC device, the leading diagonal elements would be the same, showing simultaneous modulation for both polarizations, and the off-diagonal components are negligible, indicating that the device does not convert the polarization state into an orthogonal state.

### 3.2 Planar 180° STN-LC Device

Figure 3 presents the simulation results for the planar 180° STN-LC device to further investigate its potential for optical phase modulation and polarization independence. Also shown are results for the tilted 90° TN-LC device for the purposes of comparison. The parameters used in the simulation were *K*_11_ = 11.1 pN, *K*_22_ = 6.5 pN, *K*_33_ = 17.1 pN, ε_⊥_ = 5.4, ε_∥_ = 17.4, *n*_0_ = 1.5, *n*_e_ = 1.72 and the wavelength was set to λ = 632.8 nm.

**Figure 3:**
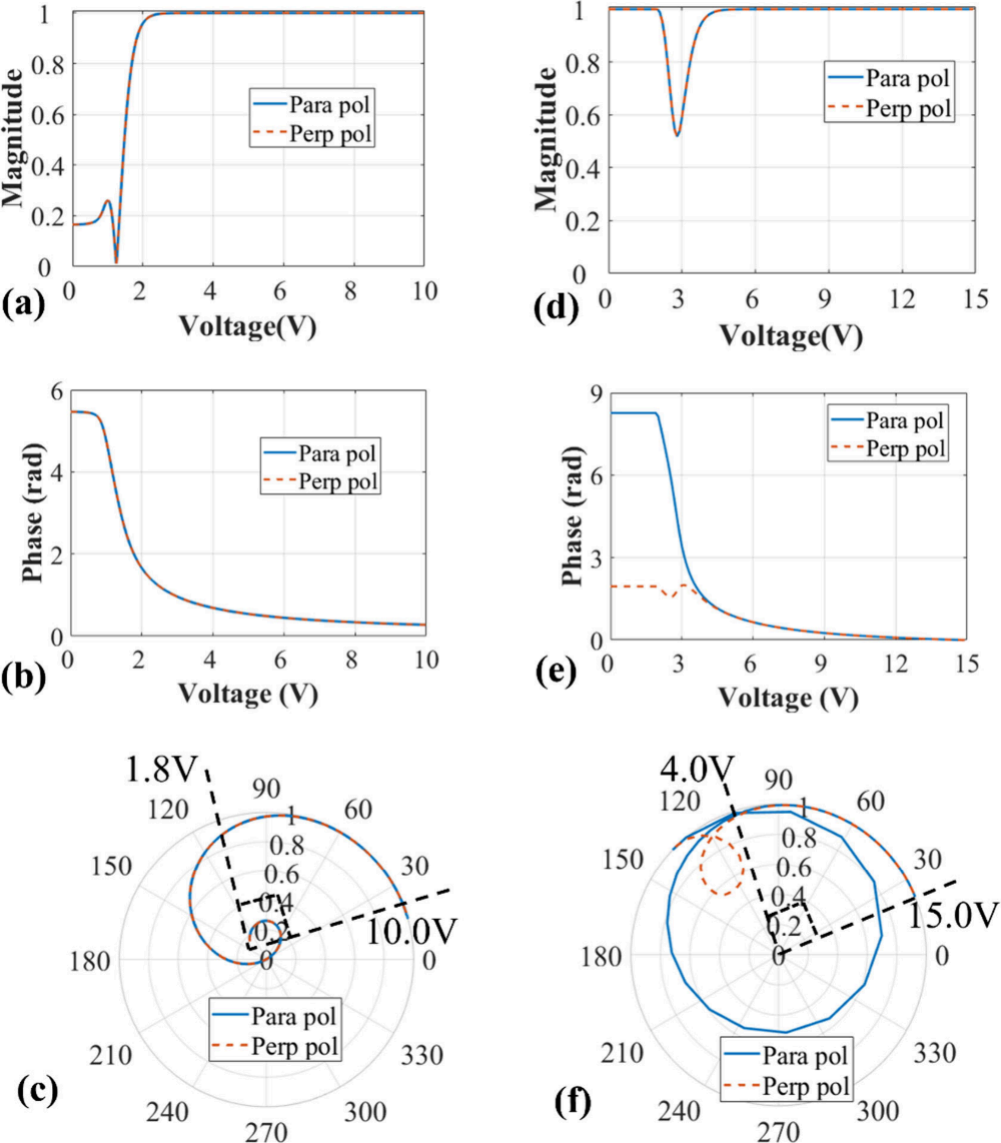
Simulation results showing the polarization independent performance for the tilted 90° TN-LC device (a–c) and the planar 180° STN-LC device switched into the T-Hs state (d–f), both with LC layer thicknesses of 5 μm. (a, d) The magnitude and (b, e) the optical phase modulation as a function of applied voltage for the two orthogonal linear polarization states. (c, f) magnitude and phase represented as a polar plot for different applied voltages and the two polarization states. In the legend, for the 90° TN-LC device, “Para pol” refers to the polarization component parallel to the alignment direction of the bottom surface (*x*-direction in Figure 1a,b)), and “Perp pol” is the polarization component parallel to the alignment direction of the top surface (*y*-direction in Figure 1a,b). For the 180° STN-LC device, “Para pol” refers to the polarization component parallel to the device’s alignment directions (*x*-direction in Figure 1m,n), and “Perp pol” refers to polarization component perpendicular to the device’s alignment direction (*y*-direction in Figure 1m,n).

To begin our discussion, we consider first the simulation results for the tilted 90° TN-LC device. Figure 3a and b present the optical magnitude and phase modulation, respectively, for a 90° TN-LC device with a thickness of 5 μm placed between parallel polarizers. The outgoing light shows the same behavior for the two orthogonal polarizations. The results indicate a relatively low magnitude when the voltage is below 2.0 V_rms_, reaching an ideal magnitude of 1 when the voltage exceeds this threshold. This transition signifies a critical operational range where the magnitude values become conducive (approximately 1) for polarization independent phase modulation capability. Here, a magnitude of 1 corresponds to 100% transmittance. For voltages below 2.0 V_rms_, the TN-LC device exhibits polarization transformation behavior, where the amplitude and phase are coupled. As the applied voltage increases from 2.0 to 10.0 V_rms_, the polarization independent optical phase modulation of this TN LC device changes from 1.5 to 0.25 rad. Within this voltage range, the liquid crystal directors become perpendicular to the surfaces, and the two surface boundary layer regions are effectively decoupled.

Simultaneous consideration of magnitude and phase modulation is vital for comprehensive analysis of optical phase modulators. To facilitate this evaluation, we employ the use of a polar plot, which offers a useful visualization where the radius corresponds to the magnitude, and the polar angle represents the achieved phase modulation. Figure 3c shows that the TN-LC device can attain about π/2 polarization independent phase modulation as the applied voltage changes from 1.8 to 10.0 V_rms_. A noteworthy aspect of this observation is the consistent phase modulation between the two orthogonal directions, which indicates that the device exhibits the same optical phase modulation characteristics for both the *x* and *y* polarized incident light simultaneously. This highlights the device’s polarization independent characteristics at high voltages. The corresponding LC director distribution was shown previously in Figure 1a–d.

We now move on to the simulation results for the planar180° STN-LC device when switched into the T-Hs state. Figure 3d and e plot the computed optical magnitude and phase modulation results of the device placed between two parallel polarizers, respectively, and Figure 3f presents these combined results as a polar plot. As the voltage changes from 4 to 15 V_rms_, the two polarization curves are consistent, and the magnitude approaches 1, indicating decoupling between the amplitude and phase performance. Within this operational range, the device exhibits successful polarization independent optical phase modulation of π/2 when switched in the T-Hs state. The corresponding LC director distribution was shown previously in Figure 1m–p.

In practical applications, a planar 180° STN-LC device with a thin LC layer thickness, such as the 5 μm thick device in Figure 3d–f, is incapable of achieving a polarization independent T-Hs state. Within a constrained space, the LC director in the bulk remains in a continuous twisted condition, instead of forming decoupled boundary layers along the *x* and *y* directions necessary for phase modulation during the switch “on” and “off” switching processes. Consequently, the continuously twisted director profile changes the polarization of incident light to its orthogonal direction, undermining the polarization independent characteristics. To address this issue, the thickness of the planar STN-LC device must be increased, which enables the boundary layers to be decoupled in the T-Hs state during the dynamic switching process, facilitating polarization-independent performance, as well as sufficient optical phase modulation.

To demonstrate the enhanced phase modulation capability, Figure 4 presents the phase modulation results for planar STN-LC devices with a thickness of 30 μm. The applied burst driving voltage is shown in Figure 4a. The results correspond to the transmission mode, where light passes through the device once, as illustrated in Figure 4b–d. The polar plot in Figure 4b highlights the phase modulation characteristics of the T-Hs state within a specific voltage range, where the magnitude approaches an ideal value of 1, and the two polarization curves remain consistent, indicating polarization independence. Notably, the device achieves a full 2π phase modulation as the applied voltage increases from 4.7 to 15.0 Vrms. The dynamic performance, shown in Figure 4c, reveals that the device attains an optical phase modulation of 1.21 rad within 1 ms during both the switch-on and switch-off processes. Figure 4d presents a polar plot that demonstrates the behavior of the device in the relaxation process from 29 to 47.2 ms. The results indicate that the transmittance remains close to 1, confirming stable polarization-independent performance. Furthermore, the phase modulation curve exhibits a half-circle trajectory, corresponding to the π phase modulation achieved in 5.3 ms, and a full-circle trajectory, illustrating the completion of 2π phase modulation in 18.2 ms.

**Figure 4:**
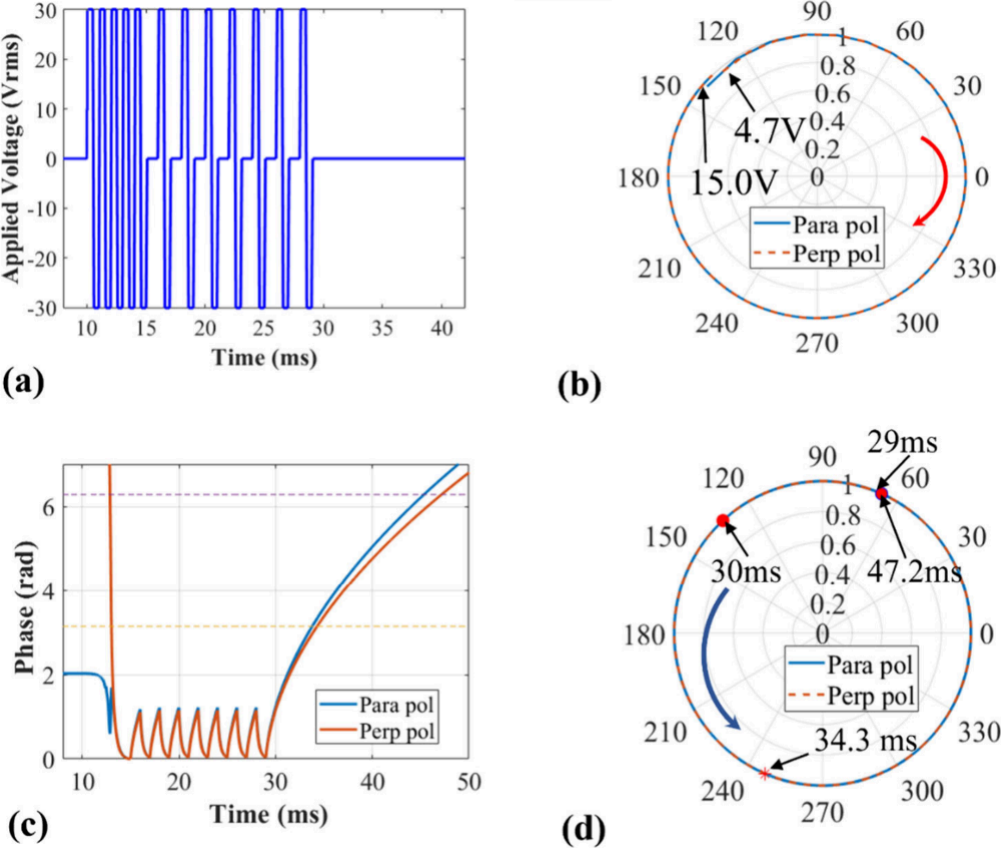
Simulation results demonstrating the polarization-independent performance of the planar 180° STN-LC device in the T-Hs state with an increased thickness of 30 μm. (a) Waveform of the burst driving voltage including modulation inside and (b) phase modulation performance as a function of applied voltage, ranging from 4.7 to 15.0 V and (c) dynamic response under a burst voltage of 30 V_rms_. The two horizontal dashed lines indicate π and 2π phase modulation, respectively, and (d) polar plot representation of the dynamic performance from 29 to 47.2 ms, extracted from (b). In the legend, “Para pol” refers to the polarization component parallel to the device’s alignment direction (*x*-direction in Figure 1m,n), and “Perp pol” refers to polarization component perpendicular to the device’s alignment direction (*y*-direction in Figure 1m,n).

These results confirm that the device maintains polarization independence in the T-Hs state while being capable of achieving nearly full 2π optical phase modulation, albeit with an extended response time. Thus, while increasing the device thickness enhances phase modulation, it introduces trade-offs, as thicker devices require higher driving voltages and exhibit slower switching speeds in certain voltage regimes.^31^

## 4 Experiment Results and Discussion

Various techniques have been developed to dynamically measure the time-resolved phase of analogue LC optical phase modulators operating at high frame rates (up to 1 kHz) with exceptional resolution.^29,32,33^ To experimentally verify the polarization independence, fast switching, and phase modulation of the T-Hs state in a planar180° STN-LC device, a phase-shifting Mach–Zehnder interferometer was assembled. Phase-shifting interferometry (PSI) is a well-established and widely used technique due to its high accuracy.^34,35,35^

As shown in Figure 5, light from a helium–neon laser with wavelength of λ = 633 nm (Thorlabs, HNL050L) passed through a nonpolarizing beam splitter, which divided the light into two paths: the signal path and the reference path. The LC device was placed in the signal path between two parallel polarizers and driven by a function generator (Multicomp PRO, MP750510). By applying a periodic burst voltage driving waveform, the device was driven into the T-Hs state, and the phase was modulated with 1 ms pulses, which was then repeated every 1 s. The reference path contained an Acoustic-Optic Frequency Shifter (AOFS), which can produce a continuous phase shift with a frequency of 40 MHz. Then two paths of the beam are combined by another nonpolarizing beam splitter and received by the photodiode (Thorlabs, DET10A/M, Si biased detector) after two reflections from the mirrors. As a result, the signal received by the photodiode contains both the optical phase modulation induced by the LC device and an additional 40 MHz phase shift introduced by the AFOS. To evaluate the polarization-independent performance of the LC device, it is initially placed between two parallel polarizers to assess its response to incident light aligned with its alignment direction. Subsequently, the LC device is rotated by 90° to measure its response to light from the orthogonal direction. If the LC device exhibits the same response in both directions, it can be considered polarization independent. This approach ensures high measurement efficiency as losses are primarily limited to the interferometric system and minimal losses from the optical components. Additionally, to ensure measurement accuracy, the interferometric system is rigorously calibrated. The designed PSI enables the single-shot acquisition of interference data, effectively minimizing the impact of external environmental noise and other disruptive factors.

**Figure 5:**
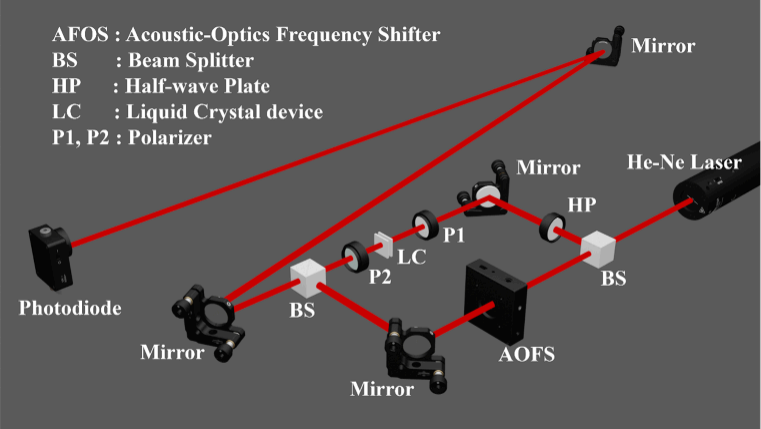
Measuring the optical phase modulation of a planar 180° STN-LC device in the T-Hs state. A phase-shifting Mach–Zehnder interferometer was used to measure the phase modulation. One path of the interferometer contained the LC device to produce the phase delay to be measured and a half-wave plate to rotate the polarization to match that produced by the AFOS placed in the other path to produce a 40 MHz continuous phase shift.

To extract the optical phase modulation information from the received signal, a Fast Fourier Transform (FFT) method was employed,^36,37^ which can efficiently determine the phase information from the signal by converting the time-domain signals into complex spectra in the frequency-domain. The received signal contained both the LC device’s phase modulation and a 40 MHz continuous phase shift, while the reference signal only contained the latter phase component. Therefore, the phase modulation of the device was obtained by subtracting the phase of the received signal from that of the reference signal. To ensure accuracy, it was crucial to use phase unwrapping when dealing with values exceeding the [−π, π] range. This approach using FFT enables efficient extraction of the phase information from the detected signals. Additionally, applying FFT enhances the resolution of the phase modulation results by using a high sampling rate of the original data. This approach allows for a precise representation of the phase modulation with very short time intervals.

Figure 6 presents the experimental results for the performance of a planar 180° STN-LC device switched into the twisted Hs state at room temperature. The device, with a thickness of 8.9 μm, can be driven into the transient T-Hs state by using a burst driving voltage, enabling the investigation of its performance within the limited existence of the transient state. As shown in Figure 6a, the designed voltage waveform consists of two stages: a priming stage and a modulation stage. During the priming stage, a 1 kHz square wave at 30 V_rms_ is applied for 5 ms (10–15 ms) without modulation, ensuring that the device fully transitions into the T-Hs state. This is followed by the modulation stage, where the driving signal alternates between an “on” state for 1 ms and an “off” state for 1 ms. Subsequently, a relaxation stage followed with no voltage signal applied, which enabled the device to fully recover to the ground state.

**Figure 6:**
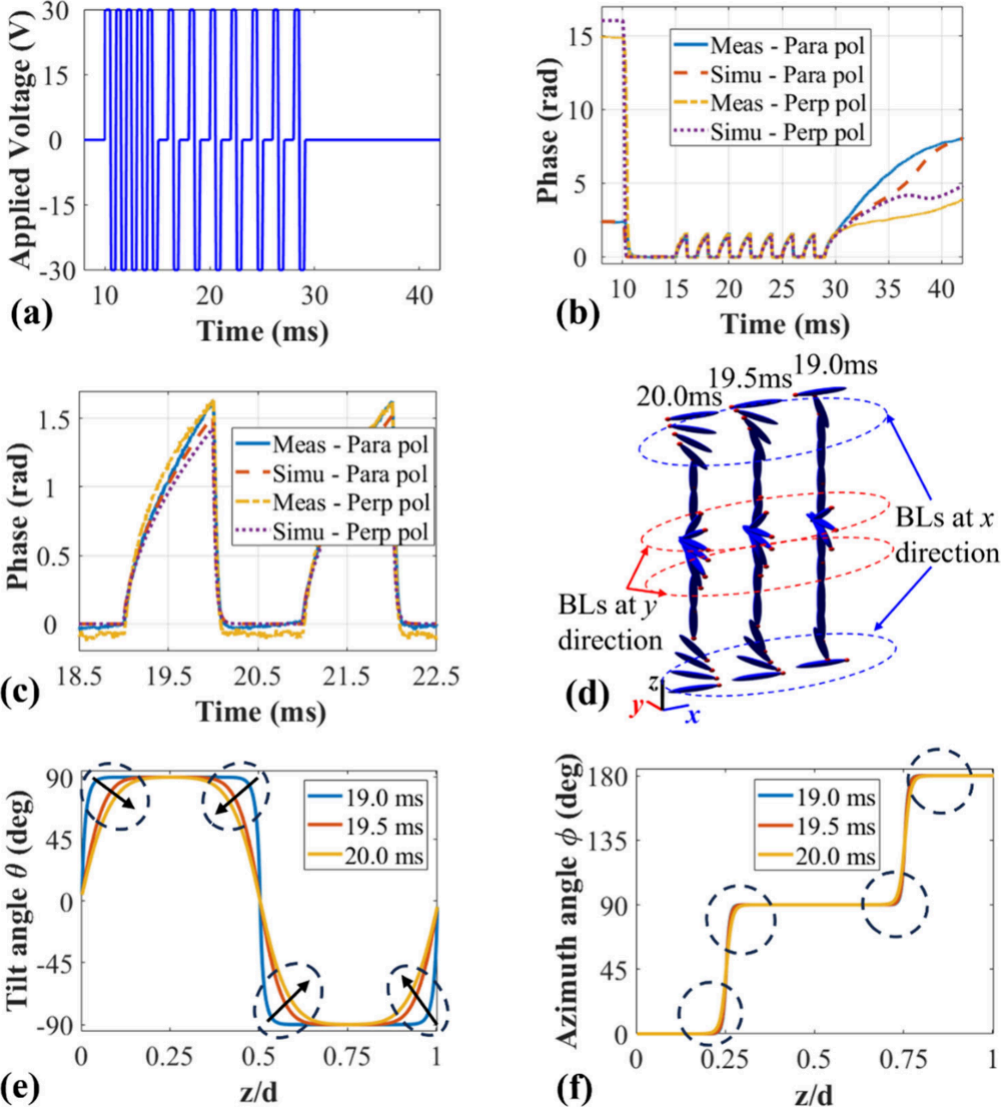
Dynamic optical phase modulation of a planar 180° STN-LC device switched into the T-Hs state at room temperature. (a) The burst driving voltage applied to the device, which consisted of a 20 ms operating period repeated every 1 s. (b) The measured and simulated optical phase modulation for two polarization states of the device: parallel (“Para pol”) and perpendicular (“Perp pol”) to the surface alignment. (c) Enlarged section of (b) to illustrate the consistency between experiments and simulations. (d) Illustration of the director distribution during the relaxation process in 1 ms (BL indicates boundary layers) and corresponding plots of (e) the tilt angle and (f) the azimuth angle of the director orientation at different times during the switching process.

Figure 6b and c depict the measured and simulated optical phase modulation results, respectively, for both polarizations extracted for a single-pass configuration (i.e., light passed through the device only once in transmission during the measurement) for the experimental device described in Section 2. During the voltage-on periods (e.g., 16–17, 18–19, and 20–21 ms), the device rapidly transitions into the T-Hs state within approximately 0.15 ms, leading to a nearly flat phase response curve. This rapid switching is attributed to the high voltage amplitude, which induces an almost instantaneous transition to the T-Hs state. Conversely, during the voltage-off periods (e.g., 15–16 ms, 17–18 ms, 19–20 ms, etc.), the relaxation process is significantly slower due to the viscosity of the LC, resulting in a gradually changing phase response curve. Under these conditions, the device achieves a $\frac{\pi }{2}$ phase shift within 1 ms for both polarizations in a single-pass setup, confirming its polarization-independent characteristics. With a four-pass configuration, the device achieves a full 2π phase modulation with polarization-independent performance within 1 ms during the relaxation process. The consistency between the measurement and simulation results further validates the accuracy of the simulation model as well as our understanding of the switching process. As indicated in Figure 4, the 30 μm thick LC device achieves 2π phase modulation within 5.3 ms in reflection mode and 18.2 ms in transmission mode during the relaxation period under a voltage of 30 V_rms_.

For future applications, the device can switch between multiple phase levels while maintaining stability within a $\frac{\pi }{2}$ range for extended time duration in the existence of the T-Hs state. The requirement for a blackout period (standard for all phase modulator applications) when switching between two phase levels within a π/2 phase is 1 ms for this device. Additionally, the 50% duty cycle for the switch-on and switch-off states can be modulated through the control scheme. It is worth noting that the device’s intrinsic twisted structure makes it sensitive to the illumination angle, which may limit its applicability as a phase modulator in scenarios requiring large-angle incidence. However, in most applications, where the incident light is predominantly normal to the surface, this angular dependence has a minimal impact on performance. Moreover, the burst driving scheme restricts its applicability in matrix-addressed systems. A potential solution to stabilize the transient T-Hs state involves creating a thin polymer layer at the device’s midplane using direct laser writing techniques.

Figure 6d presents the dynamic director distribution during the 1 ms relaxation process based on the simulation insights. The boundary layers in the *x* and *y* directions grow over time and are decoupled from the bulk. The device must be sufficiently thick to allow these boundary layers to remain decoupled, which is crucial for maintaining polarization-independent performance. As shown in Figure 6b between 29 and 31 ms, the device begins to exhibit polarization dependence at approximately 30.4 ms, which is around 1.4 ms into the relaxation process. At this point, the phase modulation reaches its maximum polarization-independent value of approximately 1.76 radians, after which the curves for parallel and perpendicular polarizations begin to diverge. This behavior is intrinsic to the twisted LC structure, where prolonged relaxation causes the boundary layers to couple with each other, leading to the observed polarization dependence. It is expected that using a thicker LC layer (greater than 8.9 μm) and applying specific voltages could enable greater polarization independent phase modulation, ideally achieving a full 2π phase shift, as predicted in Figure 4. To achieve this goal in a single-pass configuration within the time domain, potential strategies include: (1) increasing the LC layer thickness to expand the phase modulation range while preserving polarization independence and (2) optimizing the LC material properties, such as adjusting the viscosity or elastic constants, to facilitate faster switching and achieve greater phase modulation under similar driving conditions.

Figure 6e,f further illustrate the dynamic director profiles during this switching process based on simulation insights. The tilt angle θ changes at different time points, which reflects the growth of the boundary layers. Meanwhile, the azimuth angle, φ, remains stable during the process, which signifies the decoupling of the four boundary layers. This stability and the decoupling imply that the LC director responsible for phase modulation maintains a consistent orientation without twisting during the switching time. Since a twisted LC director typically contributes to polarization transformation, its absence ensures polarization independent phase modulation. This characteristic ensures successful polarization independent phase modulation with the transient T-Hs state of the planar 180°STN device.

Lastly, Figure 7a and b depict polar plot representations of the simulated dynamic switching process for the 8.9 μm device during the 1 ms “on” and 1 ms “off” cycles, respectively. The plots consistently maintain a magnitude of 1, indicating polarization independent of phase modulation. The similarity between the “switch-on” and “switch-off” processes suggests a smooth growth and compression of the decoupled boundary layers. Additionally, the consistent phase modulation curves for both polarizations confirm a polarization independent phase modulation of π/2 within 1 ms with no amplitude modulation present.

**Figure 7:**
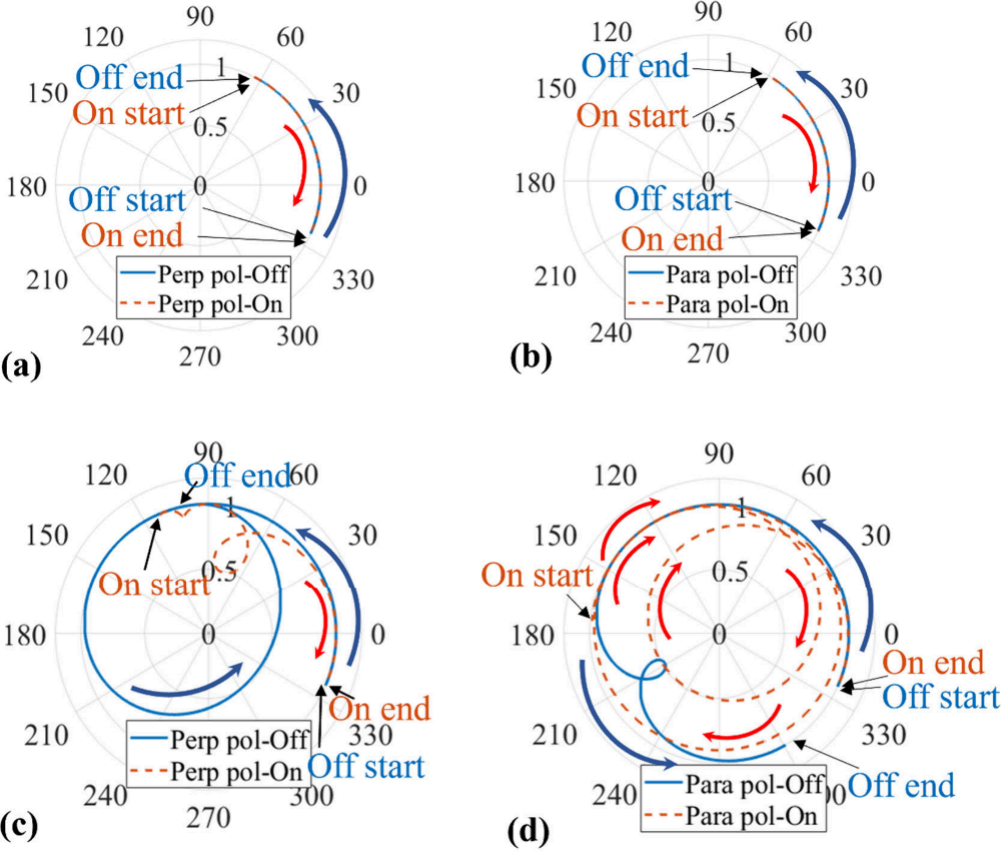
Simulated optical phase performance for the “switch-on” and “switch-off” processes in 1 ms for polarizations perpendicular (a) and parallel (b) to the surface alignment for the 8.9 μm planar 180° STN LC device. The simulated optical phase performance for the switch-on process and switch-off process in 15 ms for polarizations perpendicular (c) and parallel (d) to the surface alignment.

Additionally, Figure 6b reveals an interesting observation that at *t* = 29 ms, the two polarization curves remain consistent for only 1.2 ms before diverging. This suggests that this device can maintain polarization independence for just 1.2 ms after the 30 V_rms_ voltage is removed. To explore this further, Figure 7c,d simulate the “switch-on” and “switch-off” process lasting for 15 ms. The results show that the start point of the “switch-on” process is aligned with the end point of the “switch-off”, as the LC director in the device completely relaxes at this time. Moreover, in the middle of the switching process, the curves for the switch-on are quite different from those of the “switch-off”. This discrepancy can be attributed to the growth of boundary layers during this period and the onset of coupling to the bulk, resulting in a continuous structure with the LC director orientation angle φ changing along different paths during the “switch-on” and “switch-off” processes.^38^ This inconsistency leads to varying phase modulation curves, indicating inconsistent polarization-dependent performance during this switching period.

## 5 Conclusions

In this paper, we have investigated the potential for achieving polarization independent characteristics using twisted nematic LCs and highlight the potential of planar 180° STN devices for use as polarization independent optical phase modulators. It is shown that to achieve polarization independent behavior requires the formation of the so-called twist symmetric H state (T-Hs) using a burst driving voltage. The T-Hs state is distinguished by the formation of central boundary layers that orient perpendicular to the alignment direction at the substrate surfaces. Together, these two newly formed LC boundary layers, along with the two layers formed originally along the alignment direction, enable the device to modulate light for two perpendicular polarizations simultaneously, thus achieving optical polarization independence.

Experiments were carried out using a phase-shifting Mach–Zehnder interferometer, which enabled time-dependent optical phase variation to be obtained by employing FFT analysis. The device used in the experiments was a nematic LC Fréedericksz device with an LC layer thickness of 8.9 μm, filled with a long-pitch chiral nematic LC mixture to form a planar 180° STN device. The experimental results revealed that this device switched into the T-Hs state can achieve polarization independence as well as π/2 optical phase modulation in 1 ms under a burst voltage of 30 Vrms at room temperature in a transmission configuration. The measurement results show good consistency with the simulation results that were based upon a combination of the Ericksen–Leslie continuum equations to determine the dynamic director profile and then the Jones calculus to compute the phase and polarization properties.
